# We Can, but Should We?

**DOI:** 10.3389/fped.2016.00001

**Published:** 2016-01-19

**Authors:** Robert Brett Kelly

**Affiliations:** ^1^Division of Critical Care, Department of Pediatrics, Mattel Children’s Hospital UCLA, David Geffen School of Medicine at UCLA, Los Angeles, CA, USA

**Keywords:** pediatric, cardiopulmonary resuscitation, resiliency, burn-out, global health

We can, but should we? After recently reflecting upon our profession as pediatric critical care physicians, I concluded that this one complex question gets asked many times throughout the day – or at least it should. A recent report discussed an emerging paradigm in pediatric critical care medicine (1). With more and more chronic patients filling our pediatric intensive care unit (PICU), perhaps we should all stop and take a breath. Are we here to cure, or are we here to heal?

My views regarding life and death have certainly evolved over the last 10 years – and I think this evolution has been for the better. Although I hesitate to preach how we, as pediatric critical care physicians, should interpret our hospital work given our varied cultural, familial, and experiential backgrounds, perhaps I can simply suggest that we need to. We are, after all, human beings who have emotions, relationships, goals, and, hopefully, a sense of vocation.

Resiliency has become the new buzzword. Our pediatric residents are now embarking on resiliency training utilizing the Families OverComing Under Stress (FOCUS) program (2, 3). I have joined them. I recently filmed a piece chronicling my career and personal life particularly focusing on my time as an intensivist. Even though I tell my story, we want trainees to see that processing their own professional and personal timelines can put their career in perspective. Whether you enjoy watching a professional sports team or going out on date night to deal with the stresses of your professional life, that is not the point. The point is we need to model a healthy lifestyle for our trainees.

What has recently concerned me, however, is the unhealthy, yet popular, strategy of completely separating one’s work life from one’s personal life. When we speak of work–life balance, the important term is “balance.” Just look at the medical students we teach. Over a 3-day period, I recently taught the basics of cardiopulmonary resuscitation (CPR) to a group of 172 medical students. My colleague and I in rapid-fire 20-min sessions gave them just a taste of what we routinely do in the PICU. I cannot tell you how struck I was to see and hear their enthusiasm for medicine and their career choice. I hope they saw the same in me, but I know there are plenty in our field who are burnt out and should never teach such impressionable learners without first processing their own careers and lives. Once, they were just like these students. What happened?

I am not suggesting that we keep our pagers on around the clock or our work email on push. We all need our rest and recovery. We need to enjoy a good movie. We need to see family. We need to catch up with friends. But we also need to recover our vocation. Our vocation is to heal children. Our vocation is to teach. Our vocation is to speak up for those who need our help. Our vocation is to accept death and suffering just as we accept life. Here is the critical point. Life cannot be compartmentalized. Discomfort, pain, and death are part of life, and divorcing the tragedies we see in the PICU from our personal life is not healthy. We need to accept this and not live our personal lives in the opposite extreme.

Perhaps that is why we are seeing more burn-out. Yes, the stresses of medical insurance, access to care and poverty are real, but who wants to go to work if work is tragic and home is debauchery – or at least fantasy? I fear this has created a culture of clocking in and clocking out because no one is allowed to infringe on my pleasure – ever.

We are allowing our field to become overrun by protocols and technology. We are doing less doctoring – perhaps because we can. There is a life-support machine for nearly every organ. Why not use them? There is a “proven” protocol for nearly every malady out there. Why not try them? Our families are asking for them. How can we say, no? Starting some heroic measure or intervention is easy. Forget about discussing the merits of such an intervention. I can leave the discussion regarding its stoppage to my colleague on the next shift. I have plans after work. I can justify my actions because my family “wants everything done.” Well, as a nursing colleague flippantly said to me recently, “I want a pony, too,” when we were discussing this all too common edict.

At a medical conference I once attended, an astute professional got up to discuss just this command and how we cringe. I think every physician cringes when they hear these words. We should not. As this gentleman said, the correct answer to such an imposition is, “Everything includes comfort care, too.”

Why do we cringe? I postulate that, deep down, we know the answer. We know that we were called to heal, but now we cannot let go. We have a machine. We have a protocol. We cannot give up. We have power at our fingertips. But we cannot allow the ultimate pain and discomfort of death to become part of the conversation. Perhaps this is because our personal lives must always be comfortable, too. I think our patients’ parents share our view.

I know we can all think of that one family who stopped us in our tracks when they said no to us – no to dialysis, no to intubation, no to surgery, and no to transplantation. We have all shared these stories with our colleagues. “Can you believe they said no?” Why is this a special event in our professional lives? Why did we feel the need to tell our colleagues? Perhaps because this was a refreshing view – a rare voice crying out for comfort. Can you remember how satisfied you were to care for this patient and their family? Because, for this rare moment, you were a complete physician. You provided comfort. You were allowed to make someone’s life a little more humane. Preserving life at all costs is not what we were called to do. We were called to heal.

I came across my medical school’s guiding principle recently, *cura personalis*. The translation from Latin is care for the entire person. I remembered hearing that principle when I attended Georgetown University, but I know I did not fully appreciate its meaning until now. My fellowship research had focused on extracorporeal cardiopulmonary resuscitation (ECPR) (4). How could practitioners identify patients during CPR who would survive ECPR? I examined objective data. What I could not examine was how many physicians said no to even considering this heroic intervention. Who were these physicians? How many were there? Now we should ask, can we be one of them, ourselves?

My international medical work in Mozambique and Peru has focused on teaching pediatric CPR skills. ECPR is not an option in these settings. Here, suffering and death are a part of life. Families grieve, and physicians comfort. There are no heroic demands made. We should be spending resources on making opioids available in developing countries to alleviate suffering. We likely should be counseling and debriefing our global health trainees who return to the expansive medical resources of developed countries. How do our trainees adjust? What do they learn from their experiences? How are their careers shaped by these missions? A recent study by Balmer and colleagues touches on this global health reality and need (5). Perhaps we need to send more staff abroad – including nurses, respiratory therapists, and administrators – to refocus our priorities. Such work for me has shaped my perspective in recent years. I no longer look at ECPR in objective terms. We can, but should we?

We need to emphasize the word, balance, in our daily lives. Emotions are a part of life, and we must find meaning in what we do at work and at home. Taking time to reflect on both the good and the bad is healthy. We need to teach not only our trainees but also our seasoned colleagues how to do this. Modeling can be helpful. Writing pieces such as this one can be helpful. In my opinion, you should leave the field if you cannot or refuse to do this. We need to rekindle that enthusiasm I saw in my medical students. We need to revisit the meaning of vocation. We need to stop compartmentalizing our emotions and experiences.

By no means should we throw in the towel when the going gets tough. Do not take the easy way out. If you truly think a therapy could help, by all means try it. We need to push the envelope sometimes, but I would hazard a guess that we all know when that time has passed. Embrace it, heal, and provide comfort. *Cura personalis*. Perhaps that person should be you, first.

## Author Contributions

Dr. RK is the sole author of this opinion piece, and the opinions expressed are his alone and not necessarily the views of the David Geffen School of Medicine at UCLA or Mattel Children’s Hospital UCLA.

## Conflict of Interest Statement

The author declares that the research was conducted in the absence of any commercial or financial relationships that could be construed as a potential conflict of interest.
